# Trastuzumab Deruxtecan as a Salvage Therapy after Disitamab Vedotin Failure in HER2 Altered Solid Tumors: A Preliminary Real-world Comparative Study

**DOI:** 10.12669/pjms.42.4.14485

**Published:** 2026-04

**Authors:** Lihong Wang, Xuanye Lian, Qijing Guo

**Affiliations:** 1 Lihong Wang, Ph.D., Department of Oncology, Air Force Medical Center, PLA, Beijing 100142, China; 2 Xuanye Lian, Department of Oncology, Air Force Medical Center, PLA, Beijing 100142, China; 3 Qijing Guo, Ph.D., Department of Oncology, Air Force Medical Center, PLA, Beijing 100142, China

**Keywords:** Antibody-drug conjugates, Adverse event, Human epidermal growth factor receptor-2, Insertion mutation, Overexpression

## Abstract

**Background & Objective:**

To compare the efficacy and safety of two human epidermal growth factor receptor 2 (HER2)-targeting antibody-drug conjugates (ADCs), disitamab vedotin (RC48) and trastuzumab deruxtecan (DS-8201), in patients with the HER2 altered solid tumors.

**Methodology:**

We conducted a preliminary real-world comparative study, which included a case of a patient with HER2 exon 20 insertion mutated lung adenocarcinoma and a retrospective analysis of 18 patients treated at Air Force Medical Hospital, PLA, Beijing between 2021 and 2025. Patients received either RC48 (n=12) or DS-8201 (n=6). The primary endpoints were objective response and adverse events, evaluated using RECIST 1.1 criteria and standard toxicity assessments.

**Results:**

The case patient exhibited primary resistance and severe gastrointestinal toxicity to RC48 but achieved partial remission (PR) with DS-8201. In the cohort analysis, DS-8201 demonstrated a significantly superior PR of 66.67% compared to 8.33% for RC48 (P = 0.022). The adverse event profiles differed notably: DS-8201 was primarily associated with elevated transaminases and fatigue, while RC48 more frequently caused myelosuppression and hyperbilirubinemia.

**Conclusion:**

DS-8201 demonstrates potential as one of the effective salvage therapies following RC48 failure in HER2 altered solid tumors, showing significantly better disease control and a distinct, manageable toxicity profile. These findings highlight the importance of selecting personalized ADCs based on molecular subtypes and toxicity factors and provide a basis for future, larger-scale prospective studies.

## INTRODUCTION

The human epidermal growth factor receptor 2 (HER2/ERBB2), a member of the ERBB receptor tyrosine kinase family, is a ligand-independent receptor that activates downstream signaling pathways such as PI3K/AKT and RAS/MAPK through heterodimerisation with other ERBB receptors.^1^,^2^ These pathways critically regulate cellular processes, including angiogenesis and tumor cell proliferation, survival, and migration. *HER2* mutation, protein amplification and overexpression lead to continuous activation of the signaling pathway, which drives the behavior of many solid tumors.^3^ The *HER2* mutation rate in the Chinese population with non-small cell lung cancer (NSCLC) is 2%–4%.^4^ Most *HER2* mutations occur in the tyrosine kinase domain (85%). Exon 20 insertions are the most common *HER2* mutations; A772_G775dup insertions account for 55% and G776delinsVC insertions account for 8.3% of *HER2* mutations.^5^ HER2-targeted antibody–drug conjugates (ADCs) or tyrosine kinase inhibitors (TKIs) are mainly used for patients with tumors with *HER2* mutations and amplifications.

HER2 is overexpressed (defined by a immunohistochemistry score of 3+/2+ with positive findings on fluorescence *in situ* hybridization) in 15%–20% of breast cancers, 8%–20% of stomach/gastroesophageal junction cancers, 3%–5% of colorectal cancers (rising to 5-8% of *KRAS*/*NRAS*/*BRAF* wild-type cases), 2%–4% of NSCLCs, 5%–10% of bladder/uroepithelial cancers, 5%–10% of bile duct cancers, and 5% of ovarian cancers.^6^,^8^ *HER2* mutation and HER2 protein amplification and overexpression are associated not only with tumor invasion, lymph node metastasis, and increased recurrence risk but also with cetuximab/panitumumab resistance in patients with *RAS* wild-type colorectal cancer and those with *EGFR/KRAS/ALK* mutations in NSCLC.^9^ Both large-molecule antibodies and small-molecule TKIs targeting HER2 have demonstrated survival benefits, particularly in the form of ADCs. Moreover, ADCs have been reported to reduce the risk of death in patients with HER2-positive cancers.

ADCs are formed by linking monoclonal antibodies targeting specific antigens with small-molecule cytotoxic drugs through linkers; these ADCs exhibit the powerful anti-tumor effect of small-molecule chemotherapy and the targeting property of antibody drugs. Currently marketed HER2-targeting ADCs include ado-trastuzumab emtansine (T-DM1), trastuzumab deruxtecan (T-DXd; DS-8201), and disitamab vedotin (RC48). While T-DM1 and DS-8201 utilize trastuzumab as their antibody component, RC48 employs a distinct HER2-binding monoclonal antibody. Trastuzumab carries a risk of cardiotoxicity, primarily manifesting as left ventricular dysfunction and heart failure.^10^ T-DM1, as its ADC form, may have lesser cardiotoxicity than trastuzumab because of its targeted drug delivery system. However, residual risks remain, necessitating ongoing monitoring of left ventricular ejection fraction (LVEF).^11^ DS-8201, which binds to topoisomerase I inhibitors via a cleavable linker, has shown significant efficacy in treating several HER2-positive cancers, particularly breast and stomach tumors. With regard to its cardiotoxicity, clinical trials such as DESTINY-Breast03 (NCT03529110) have revealed relatively few cardiac-related adverse events.^12^ However, cases of left ventricular dysfunction have been reported, with an incidence of approximately 1%–2%. RC48 involves different linkers and payloads (microtubule inhibitor monomethyl auristatin E [MMAE]) and is approved in China for the treatment of HER2-overexpressed gastric cancer and urothelial carcinoma. Among current publicly available data, reports of cardiotoxicity are scarce; however, clinical trial data must be scrutinized for potential individual cases.

The emergence of HER2 ADCs has revolutionized the treatment landscape for HER2-expressing solid tumors. However, the optimal sequencing of these agents and the mechanisms underlying differential responses remain poorly understood. We began our research with a case study of a patient with lung cancer who harbored a *HER2* exon 20 insertion mutation. This led to our analysis of these patients treated with HER2 ADCs at our department from January 2021 to March 2025. By comparing the therapeutic efficacy and safety profiles of RC48 and DS-8201, we aimed to provide clinical evidence supporting personalized medication strategies.

## METHODOLOGY

We collected general information about the enrolled patients, including their sex, age, occupation, smoking history, and family medical history. We also documented the incidence of disease and the treatment process of patients, including time of diagnosis, molecular expression and gene detection, drug use, efficacy evaluation, and toxic and other adverse effects.

### Ethical Approval:

This study was approved by the Ethics Committee of the Air Force Medical Center, PLA, Beijing, China (No. 2025-85-PJ01, July 18, 2025). A waiver of informed consent was granted by the ethics committee for this retrospective analysis.

### Selection of patients treated with RC48 or DS-8201:

All data were obtained from the Air Force Medical Hospital, PLA, Beijing, China. We collected clinical data from 18 patients in the oncology department, all treated between January 2021 and March 2025 with RC48 or DS-8201 or both sequentially. Patients who received RC48 during the treatment process are classified into Group RC48 (n=12) and patients who received DS-8201 classified into Group DS-8201 (n=6). The dosage of DS-8201 is 5.4 mg/kg administered once every three weeks, while the dosage of RC48 is 2.0 mg/kg administered once every two weeks. Patients were included in this study if they met all of the following criteria: histologically confirmed cancer, HER2 expression was tested, received at least one cycle of either RC48 or DS-8201, availability of complete medical records, age ≥ 18 years old, eastern cooperative oncology group performance status (ECOG-PS) of 0 to 2. Exclusion Criteria include lack of key clinical data for efficacy or safety evaluation, history of other active malignant diseases within the past five years, severe impairment of heart, liver, or kidney function. General patient information included sex, age, ECOG-PS score, combined disease, primary disease, metastasis status, previous treatments, HER2 expression level, whether anti-HER2 treatment was received, treatment efficacy, and adverse reactions (such as general malaise or LVEF changes before and after treatment). Laboratory data included white blood cell counts; hemoglobin levels; platelet counts; and albumin, globulin, alanine aminotransferase, aspartate aminotransferase, and gamma-glutamyl transferase levels. For the purpose of comparing the efficacy and safety profiles of the two HER2-targeting ADCs, patients were categorized based on the treatment they received. This approach allowed us to evaluate each drug’s performance independently. To minimize selection bias, we included all eligible patients identified through the institutional database during the study period.

### Statistical analysis:

All data were analyzed using GraphPad Prism 8.3.0 and IBM SPSS statistical analysis software. Descriptive data are presented as frequency and mean ± standard deviation (SD). Fisher’s exact test was used to compare pairwise parameters between different treatment groups. *P* <0.05 was considered statistically significant. Patients were treated at a single center and received distinct therapies (DS-8201 or RC48); therefore, the potential clustering effects were not formally modeled because of the limited sample size and distinct treatment groups. Analyses focused on group-level comparisons.

## RESULTS

### DS-8201 was effective against RC48-resistant lung adenocarcinoma with HER2 exon 20 insertion:

**Fig.1:**
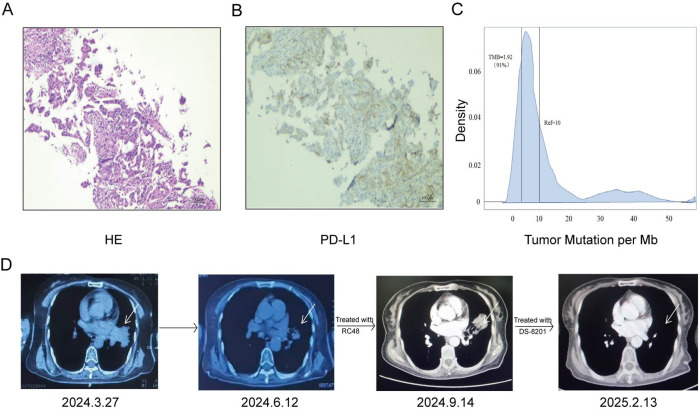
Partial pathological and clinical images of a lung adenocarcinoma with HER2 exon 20 insertion mutation. (A) Hematoxylin-eosin staining of lung biopsy specimen. (B) PD-L1 immunohistochemical staining of lung biopsy specimen (tumor proportion score, 10%). (C) Low tumor mutational burden (TMB: low). (D) According to the response evaluation criteria in solid tumors (RECIST) 1.1 evaluation criteria, the patient’s disease progressed after RC48 treatment. The efficacy of subsequent DS-8201 treatment was evaluated as maintaining partial remission.

A 54 years old non-smoking woman presented with pain in her left lower extremity. Imaging revealed occupying lesions in the left upper lung lobe and left pulmonary hilum, along with multiple brain metastases. Biopsy pathology confirmed poorly differentiated adenocarcinoma, and hematoxylin and eosin staining revealed that it was ALK-negative (Fig.1A). Next-generation sequencing revealed a *HER2* exon 20 insertion mutation (A775-G776insYVMA) with a variant allele frequency of 22.5% and PD-L1 expression (SP263; tumor proportion score, 10%; Fig.1B), low tumor mutation burden (Fig.1C), and microsatellite-stable status. In March 2024, the patient received one cycle of intravenous pemetrexed, carboplatin, and sintilimab. Treatment was discontinued because of grade IV gastrointestinal toxicity (nausea and vomiting). In early April 2024, the therapy was changed to pyrotinib. However, intolerable nausea and vomiting persisted with weight loss, prompting treatment cessation. In June 2024, the patient was received RC48 treatment. The patient tolerated the treatment poorly and severe gastrointestinal reactions, which mainly manifested as grade IV nausea and vomiting, and she lost 15 kilogram (kg) in body weight (from a baseline of 78 kg) during the treatment period. Computed tomography in September 2024 revealed a new left ischial metastasis. Partial remission was achieved with subsequent DS-8201 treatment (Fig.1D). The patient felt fatigued. The patient’s left lower extremity pain resolved completely after this treatment.

### Comparison of the clinicopathological characteristics, therapeutic effect, and adverse effects of RC48 and DS-8201:

Of these patients treated with ADCs, ten received only RC48, four received only DS-8201, and two received both sequentially. Among the patients treated with DS-8201, the primary tumors were lung cancer in three and breast cancer in three. Primary tumor sites among patients treated with RC48 were more varied: salivary gland cancer (n = 1), parotid gland cancer (n = 1), breast cancers (n = 3), pancreatic cancers (n = 2), ureteral cancers (n = 2), colon cancer (n = 1), bladder cancer (n = 1), and lung cancer (n = 1). The average age of patients treated with RC48 was 62.09 ± 7.39 years, whereas that of patients treated with DS-8201 was 56 ± 14.78 years. Sex distribution was comparable, with males comprising 25.00% (3/12) and females 75.00% (9/12) of the RC48 group compared with 33.33% (2/6) males and 66.67% (4/6) females in the DS-8201 group (Table-I).

**Table-I:** Clinical characteristics, treatment effect, and adverse events of patients treated with HER2 ADCs.

Characteristics	RC48(n=12)	DS-8201(n=6)	P value
Age(year) mean±SD^1^	62.09±7.39	56±14.78	0.309
Gender	1.000		
Male	3(25.00%)	2(33.33%)	
Female	9(75.00%)	4(66.67%)	
Concomitant disease			
Hypertension	3(25.00%)	3(50.00%)	0.344
Diabetes mellitus	6(50.00%)	1(16.67%)	0.316
Coronary artery disease	4(33.33%)	3(50.00%)	0.627
Others	3(25.00%)	1(16.67%)	1.000
ECOG-PS	1.000		
0	0(0%)	0(0%)	
1	9(75.00%)	5(83.33%)	
2	3(25.00%)	1(16.67%)	
Metastasis sites			
Lung	3(25.00%)	1(16.67%)	1.000
Liver	4(33.33%)	2(33.33%)	1.000
Brain	2(16.67%)	2(33.33%)	0.569
Lymph node	10(83.33%)	5(83.33%)	1.000
Others	4(33.33%)	1(16.67%)	0.615
HER2 status	0.062		
IHC 1+	2(16.67%)	1(16.67%)	
IHC 2+	2(16.67%)	1(16.67%)	
IHC 3+	6(50.00%)	0	
Exon 20 insertion	1(8.33%)	3(50.00%)	
Previous anti-HER2 treatment	0.214		
Yes	1(8.33%)	2(33.33%)	
No	10(83.33%)	3(50.00%)	
Treatment cycles	0.547		
1-2cycles	2(16.67%)	2(33.33%)	
3-12cycles	9(75.00%)	3(50.00%)	
Effect evaluation	0.058		
CR	0	0	
PR	1(8.33%)	4(66.67%)	0.022
SD^2^	8(66.67%)	2(33.33%)	
DCR	9(75.00%)	6(100%)	
PD	3(25.00%)	0	
Adverse events			
LVEF decreased	4(33.33%)	1(16.67%)	0.615
Fatigue	6(50.00%)	5(83.33%)	0.316
WBC decrease	1(8.33%)	1(16.67%)	1.000
Hb decrease	3(25.00%)	0	0.515
PLT decrease	1(8.33%)	0	1.000
Liver function lesion			
Transaminases increase	2(16.67%)	3(50.00%)	0.268
Bilirubin increase	5(41.67%)	2(33.33%)	1.000
gamma-GGT increase	6(50.00%)	3(50.00%)	1.000
Albumin decrease	1(8.33%)	1(16.67%)	1.000
Nausea	3(25.00%)	3(50.00%)	0.344
Oral ulcer	1(8.33%)	2(33.33%)	0.245

HER2: Human epidermal growth factor receptor 2; ADC: antibody–drug conjugates; SD^1^: standard deviation; ECOG-PS: Eastern cooperative oncology group performance status; IHC: immunohistochemistry; CR: complete remission; PR: partial remission; SD^2^: stable disease; DCR: disease control rate; PD: progressive disease; LVEF: left ventricular ejection fraction; WBC: white blood cell; Hb: hemoglobin; PLT: platelet; GGT: glutamyl transpeptidase.

Authors Contribution:

LW: Conceptualization and design. Methodology. Preparation of original draft. Accountable for the accuracy of the studyXL and QG: Data curation and acquisition, Investigation. Critical review. All authors have read and approved the final version for publication.

Hypertension (50.00% vs. 25.00%) and coronary artery disease (50.00% vs. 33.33%) were more frequent in the DS-8201 group than in the RC48 group, whereas diabetes mellitus was more predominant in the RC48 group than in the DS-8201 group (50.00% vs. 16.67%). Nine (75.00%) patients in the RC48 group and five (83.33%) patients in the DS-8201 group had an ECOG-PS score of 1. Moreover, three (25.00%) and one (16.67%) patient in the RC48 and DS-8201 groups had an ECOG-PS score of two, respectively. Both groups had identical rates of lymph node metastasis (83.33%). No notable differences were observed in other metastatic sites, such as bone, muscle, and adrenal glands. HER2 immunohistochemistry scores of 3+ were more common in the RC48 group than in the DS-8201 group (50.00% vs. 0%), whereas exon 20 insertion mutations were significantly more frequent in the DS-8201 group than in the RC48 group (50.00% vs. 8.33%).

We used the **r**esponse **e**valuation **c**riteria **i**n **s**olid **t**umors **v**ersion (RECIST) 1.1 criteria to evaluate the tumor response. The DS-8201 group demonstrated higher partial remission rates (66.67%) than the RC48 group (8.33%) but lower stable disease (SD) rates (33.33%) than the RC48 group (66.67%). The disease control rate in the DS-8201 group was slightly higher than that in the RC48 group (*P* = 0.058). Fatigue and elevated transaminase levels were more common in the DS-8201 group, whereas hyperbilirubinemia and myelosuppression were predominant in the RC48 group with no statistically significant difference. The DS-8201 group showed higher disease response and hepatic toxicity rates, higher gastrointestinal reactions, and a smaller impact on LVEF than the RC48 group.

## DISCUSSION

Our retrospective study findings initially underscore the divergent clinical outcomes and toxicity profiles of two HER2-targeted ADCs, RC48 and DS-8201. This differential response of our patients highlights the impact of structural variations between these agents on therapeutic efficacy.

RC48, which delivers a MMAE,^13^ was associated with severe gastrointestinal toxicity and rapid disease progression in this patient, whereas DS-8201, which delivers a topoisomerase-I inhibitor payload (exatecan derivative) with a cleavable linker, achieved disease control with manageable fatigue and no gastrointestinal complications.^14^ These observations were comparable to those of the cohort analysis, in which DS-8201 produced significantly higher partial response rates (66.67%) than RC48 (8.33%); however, the toxicity patterns differed (transaminase increase and fatigue vs. myelosuppression and hyperbilirubinemia). The meta-analysis of 14 studies concluded that the disease control rate of DS-8201 was higher than that of RC48.^15^ Kotteas E et al.^16^ reported that patients using DS-8201 had a 6.75% chance of developing interstitial pneumonia, but this adverse event was not observed in our cohort. The enhanced efficacy of DS-8201 may stem from its higher drug-to-antibody ratio, bystander effect, and improved penetration into the tumor microenvironment, which can overcome the resistance mechanisms linked to the payload or unstable linker systems of RC48.^17^

Our cohort data revealed the clinical significance of heterogeneity in *HER2* expression in treatment selection. Patients treated with RC48 mainly had HER2 overexpression (50%), but most patients treated with DS-8201 harbored *HER2* exon 20 insertion mutations. Tsao et al.^18^ believed that the efficacy of DS-8201 in treating breast cancers with low or no *HER2* expression is related to extracellular proteases in the tumor microenvironment, such as cathepsin L. In HER2-positive breast cancer, the antibody backbone of DS-8201 binds to the Fcγ receptor and drives antibody-dependent phagocytosis, which activates myeloid cells and further enhances the presentation of tumor antigens to CD8^+^ T cells. Cancer is associated with chronic inflammation and a series of subsequent immune responses. Conversely, the limited efficacy of RC48 in cases of *HER2* exon 20 mutations might reflect insufficient intracellular MMAE release or inadequate target engagement in specific metastatic niches based on existing literature hypotheses. The slight differences in toxicity between RC48 and DS-8201 further emphasize the need for side effect-driven individualized treatment.

### Limitations:

It includes small sample size (especially in the DS-8201 group with n=6), retrospective design, single-center nature, baseline imbalance between groups (the inherent selection bias and unmeasured confounding factors, such as reasons for treatment selection, subtle differences in performance status). The absence of serial biomarker analyses limited the mechanistic understanding of ADC resistance.

## CONCLUSION

Our findings reinforce the potential of DS-8201 as an option for treating cancers with *HER2* expression, even after RC48 failure, and highlight the irreplaceable role of ADC structural optimization in balancing efficacy and safety. In the future, researchers should prioritize prospective comparisons of these ADCs, integrating biomarker-driven stratification and exploring combinatorial strategies to prolong therapeutic durability for these molecularly defined, clinically challenging diseases.
